# Using RNA-seq to characterize pollen–stigma interactions for pollination studies

**DOI:** 10.1038/s41598-021-85887-y

**Published:** 2021-03-23

**Authors:** Juan Lobaton, Rose Andrew, Jorge Duitama, Lindsey Kirkland, Sarina Macfadyen, Romina Rader

**Affiliations:** 1 School of Environmental and Rural Science, University of New England, Armidale, Australia; 2 Systems and Computing, Engineering Department, Universidad de Los Andes, Bogota, Colombia; 3 CSIRO, Clunies Ross St., Acton, ACT Australia

**Keywords:** Biological techniques, Computational biology and bioinformatics, Ecology, Genetics, Molecular biology, Plant sciences

## Abstract

Insects are essential for the reproduction of pollinator-dependent crops and contribute to the pollination of 87% of wild plants and 75% of the world’s food crops. Understanding pollen flow dynamics between plants and pollinators is thus essential to manage and conserve wild plants and ensure yields are maximized in food crops. However, the determination of pollen transfer in the field is complex and laborious. We developed a field experiment in a pollinator-dependent crop and used high throughput RNA sequencing (RNA-seq) to quantify pollen flow by measuring changes in gene expression between pollination treatments across different apple (*Malus domestica* Borkh.) cultivars. We tested three potential molecular indicators of successful pollination and validated these results with field data by observing single and multiple visits by honey bees (*Apis mellifera*) to apple flowers and measured fruit set in a commercial apple orchard. The first indicator of successful outcrossing was revealed via differential gene expression in the cross-pollination treatments after 6 h. The second indicator of successful outcrossing was revealed by the expression of specific genes related to pollen tube formation and defense response at three different time intervals in the stigma and the style following cross-pollination (i.e. after 6, 24, and 48 h). Finally, genotyping variants specific to donor pollen could be detected in cross-pollination treatments, providing a third indicator of successful outcrossing. Field data indicated that one or five flower visits by honey bees were insufficient and at least 10 honey bee flower visits were required to achieve a 25% probability of fruit set under orchard conditions. By combining the genotyping data, the differential expression analysis, and the traditional fruit set field experiments, it was possible to evaluate the pollination effectiveness of honey bee visits under orchards conditions. This is the first time that pollen-stigma-style mRNA expression analysis has been conducted after a pollinator visit (honey bee) to a plant (in vivo apple flowers). This study provides evidence that mRNA sequencing can be used to address complex questions related to stigma–pollen interactions over time in pollination ecology.

## Introduction

To ensure yields are maximized in pollinator-dependent crops and conserve wild plants, it is essential to understand the quantity and quality of pollen transfer by pollinators^1,2^. However, the determination of pollen receipt on flowers in the field is complex and laborious^3–6^. Current methods include counting pollen grains deposited on pistils, pollen tube counts, and resulting fruit/seed set after single or multiple pollinator visits^7^. Nevertheless, most of these methods lack the fine-scale resolution to evaluate the genotype of pollen transferred and the implications for plant reproductive success and fitness^8^. Pollen grains from closely related species or different cultivars within a single species are almost impossible to identify under a light microscope^9–12^. While DNA metabarcoding has improved this resolution in many cases, pollen donor identification is of limited use unless reference libraries include all plant species from the local ecosystem. Further, metabarcoding has limited applicability in identifying the transfer of conspecific, compatible pollen onto the stigma surface^13–18^. Although counting pollen tubes can be informative to gauge the success of pollen–stigma interactions, it is limited in its capacity to identify which pollen grain donors were present in the interaction^16^.

Genetic technologies such as high throughput sequencing provide alternative options for characterizing a pollination event at the molecular level^16^. In particular, high throughput sequencing of RNA (RNA-seq) enables the characterization of transcription dynamics at the level of tissues. Therefore, the gene expression variation can be captured as a result of pollen–stigma genotype interactions^19–23^. Pollen transcriptomic analyses have been used to understand gamete development, specific pollen gene expression, pollen tube dynamics, pollen–stigma interactions, and candidate genes for self-incompatibility^22,24–26^. While gene expression profiles are able to distinguish between self and outcross pollination, the identification of pollen donors requires genotyping obtained from high throughput sequencing data. Crop cultivar genotyping is a conventional molecular tool for plant breeding programs, and molecular markers like single nucleotide polymorphisms (SNPs) are commonly used to discriminate mutants and specific cultivars alleles^27^. Previous studies have successfully used SNPs genotyping methods to differentiate pollen from the stigma tissue or the stigma response to different pollen donors^28,29^. However, this approach has rarely been validated in the field, and has not previously been used to detect and discriminate pollen cultivars, cultivar outcross pollen transfer, and natural pollination effect by bees.

The identification of pollen genotypes deposited after a given number of pollinator visits provides greater resolution for the study of plant–pollinator networks, a challenging task that traditionally relies on pollinator observations and microscopy techniques with relatively low genetic resolution^16^. Identification of the pollen donor is crucial in pollinator-dependent food crops, where cross-pollination across cultivars is required for high-quality fruit production^30^. Importantly, the determination of the compatibility of deposited pollen enables understanding of the pollinator services delivered before fruit set occurs^8^. This is advantageous for studies of pollen–stigma interactions and pollen compatibility, avoiding the need to wait for fruit set and to disentangle resource assimilation associated with pollinator effectiveness metrics.

In this study, we used mRNA sequencing in conjunction with a field experiment in commercial apple (*Malus domestica* Borkh.) orchards to test the utility of RNA-seq in detecting a successful outcrossing pollination event. Outcrossing in apple orchards is critical for producing the fruit of the ideal size and quality^30–32^. We compared gene expression, sequence variation, and fruit set resulting from single and multiple floral visits by honey bees, and experimental hand pollination treatments to understand the extent to which a successful pollination event can be detected using RNA-seq. Specifically, we ask three research questions:

1. Can cross-pollination be detected using RNA-seq? Differential gene expression was predicted in the cross-pollinated samples compared to the bagged control where access by honey bees was prevented.
2. To what extent does time since pollination impact the detection of the event? Gene expression differences were predicted to increase in magnitude with time since pollen deposition.
3. Can the cultivars of pollen donors be distinguished post-deposition? Cross-pollination was predicted to be evident from the cultivar sequence variation.

## Results

### Pollination can be detected in orchards using RNA-seq

The RNA-seq on pollen and pollinated stigmas samples resulted in more than 18 billion mRNA reads, with an average of 34 million reads per sample (Supplementary Table S1). At least 73% of reads from each sample (mean 79.67%) mapped to the apple Golden Delicious reference genome (Supplementary Figure S1 online). The reads unmapped to the apple reference genome represents the unique apple cultivar reads, and the microbes associated with environmental samples. A metagenomics analyses of a subset of reads revealed the presence of archaea, bacteria, fungi, protozoans, and specific apple viruses i.e. apple stem pitting virus, apple chlorotic leaf spot virus, and apple steam grooving virus. An additional, metagenomics analysis of the total reads identified low percentage alignments to pear (*Pyrus bretschneideri*), exhibiting sequence similarity between apples and pears. The results demonstrated that using a reference mapped base approach it was possible to select the apple reads and capture the pollination response in natural scenarios, avoiding the possible environmental and contamination noise. Significant differential gene expression (p value) was detected in all pollination treatments for thousands of genes (Supplementary Figure S2 online). Differential gene expression by false discovery test (q value) was detected only in a few pollination comparisons. The four-fold change analysis presented a set of differentially expressed genes isoforms especially in the outcross pollination treatments and the self-pollination treatments.

### Time-course of gene expression in apple pollination events

Concordant with the results of the different pollination treatments, the time-course analysis conferred a dynamic differential fold change in gene expression among all times (6, 24, and 48 h) compared to the no-pollination control (Table 1). The four-fold change analysis presented a total of 429 differentially expressed genes identified in the cross-pollination treatment at 6 h. The majority (428) of these differentially expressed genes by fold change were upregulated (Supplementary Table S2). The gene expression direction of the pollination treatments could be compared by the FPKM, q value, and the fold change distribution (Supplementary Figure S3–S4 online). At 24 h, the cross-pollination, mix-pollination, and the self-pollination treatments presented 36, 2, and 28 differentially expressed genes, respectively. At 48 h, a pattern characteristic of outcross pollination gene regulation was observed. In the cross and mix pollination treatments, the majority of the differentially expressed genes by fold change were upregulated, while in 1 V, 5 V, and self-pollination, the differentially expressed genes were down-regulated. The 48 h self-pollination treatment presented 85 down-regulated genes. All these genes were upregulated at 6 h in the cross-pollination treatment. The genes differentially expressed in all treatments, and especially those involved in the 6-h cross-pollination, were identified as genes directly related to molecular pollination tasks including, pollen development, pollen endurance, cytoskeletal dynamics, pollen tube directional growth, hormones, cell wall loosening, cell wall construction, calcium signals, allergens, kinases, RNA binding, immune defense response, and self-incompatibility system activation among other specific molecular functions. A majority of these differentially expressed genes are proteins of unknown function or without annotation (Supplementary Table S2 online).

**Table 1:** Total number of differentially expressed genes by four-fold change.

Differentially expressed genes by four-fold change	No-pollination control		
	6h	24h	48h
Hand cross (RG)	429	36	12
Hand mix (RG-GS-FJ)	1	2	24
Hand self (PL)	0	28	85
Honeybee 1 visit	0	0	19
Honeybee 5 visits	1	1	6

The gene expression evaluation of the different pollination treatments at 6, 24, and 48 h compared to no-pollination control. Hand pollination results of the cross pollination with RG, Royal gala. Hand Mix pollination with RG; GS, Granny Smith; FJ, Fuji. Hand self-pollination with PL, Pink Lady pollen. Differentially expressed genes in natural pollination treatments with single honey bee visit and five honey bee visits.

### Pollen cultivar identification from mRNA sequences

**Figure 1:**
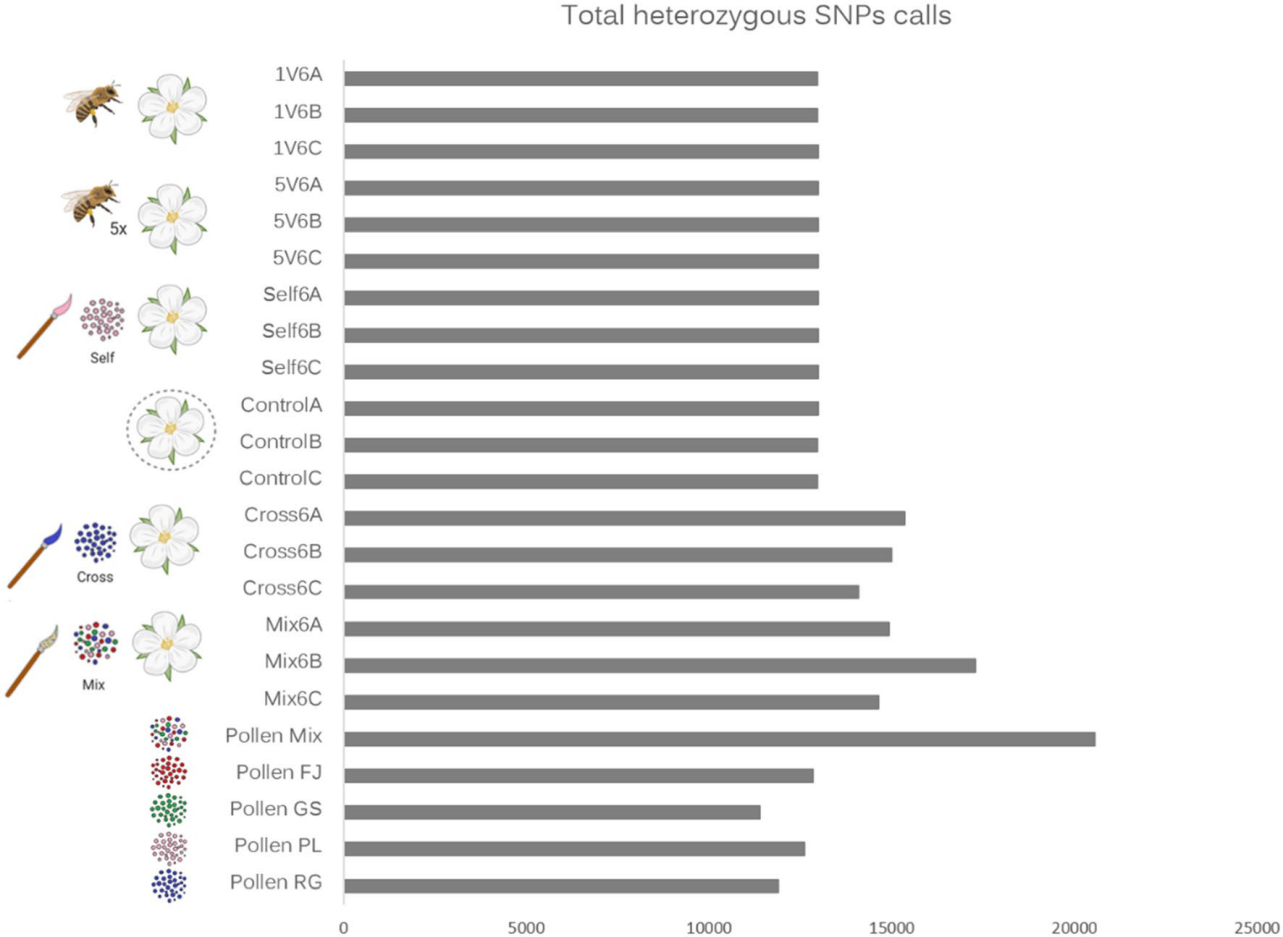
Total number of heterozygous SNPs calls. The heterozygous allele state is an indication of the variety outcross. The number of heterozygous SNPs calls of the pollination treatments and pollen varieties were obtained from the SNPs genotyping matrix (Supplementary Tables S4–S5 online). The labels denote natural pollination (honey bee), for single visit (1 V), and five visits (5 V). Bagged flowers correspond to no pollination (control). The hand-pollination treatments (brush) were self-pollination, cross pollination, and pollen variety mix pollination. The heterozygous calls were obtained at 6 h by triplicates (**A**–**C**) including the pollen cultivars and the pollen mix as references, Pollen Mix; FJ, Fuji (Red); GS, Granny Smith (Green); RG, Royal Gala (Blue); PL, Pink Lady (Pink) cultivars.

**Figure 2:**
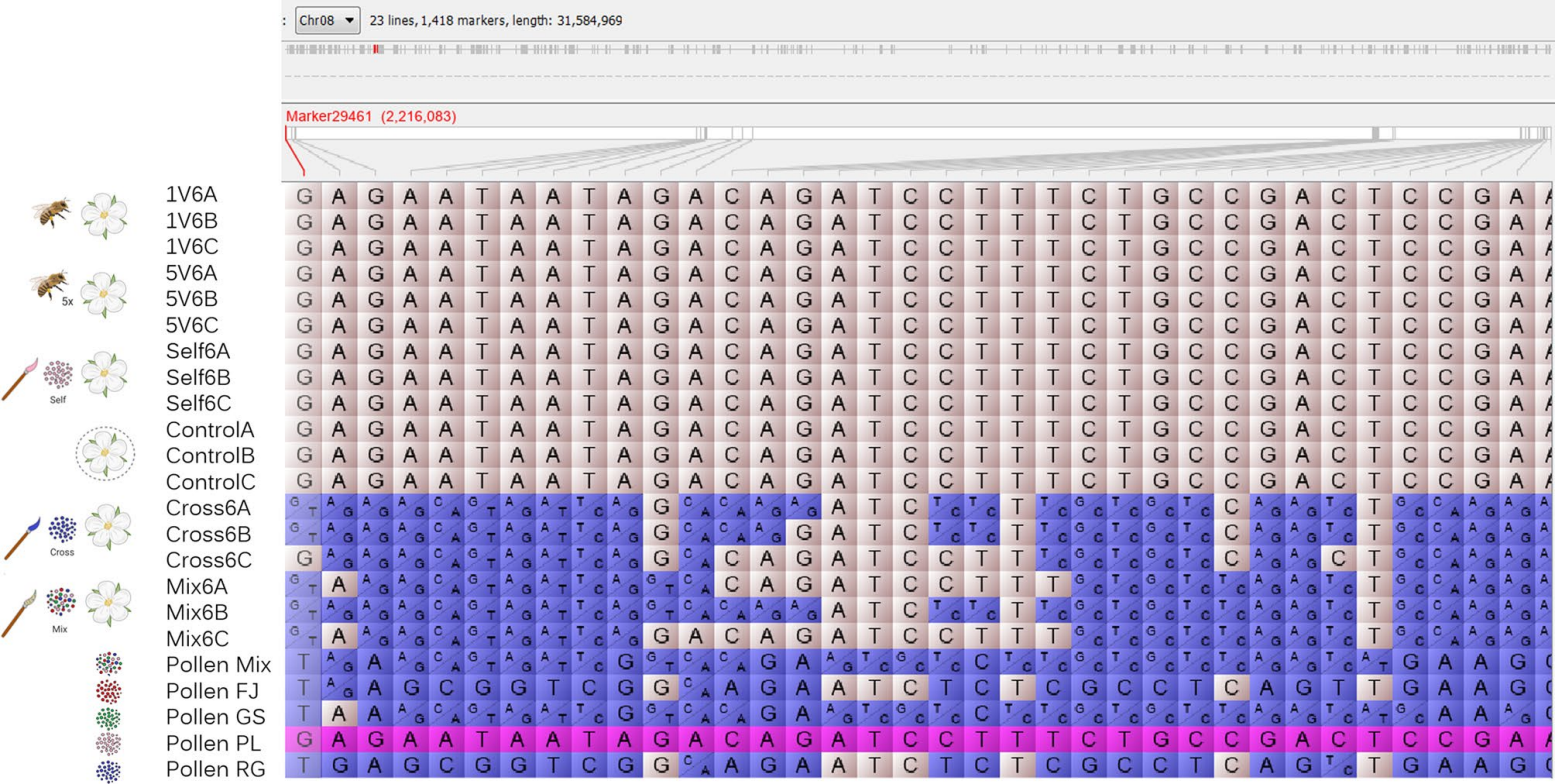
Haplotype genotype calls in pollination treatments. Haplotypes blocks differentiating the outcross pollinated stigmas with heterozygous calls, were detected along the genome, identifying the possible pollen parental donor. This particular block is located at chromosome eight (Chr 08: 2,169,950–3,276,063 bp) of the apple genome. The genotypes are colored by to similarity to the Pollen PL (purple), same genotype to pollen PL (pink), different genotype to Pollen PL (blue). The haplotype block shared in the outcross pollinated stigmas differentiate the heterozygous state to the self, control and honey bee visits homozygous calls. The labels denote natural pollination (honey bee), for single (1 V), and five visits (5 V). Bagged flowers correspond to no pollination (control). The hand-pollination treatments (brush) were self-pollination, cross pollination, and pollen variety mix pollination. The genotype calls were obtained after 6 h in triplicates (**A**–**C**) including the pollen cultivars and the pollen mix as references, Pollen Mix; FJ, Fuji (Red); GS, Granny Smith (Green); RG, Royal Gala (Blue); PL, Pink Lady (Pink) (Supplementary Table S4 online).

To assess whether pollen from different cultivars could be identified from RNA-seq data, we used SNPs in pollinated apple stigmas and pollen samples across four different cultivars: Pink lady (PL), Royal Gala (RG), Granny smith (GS), Fuji (FJ), and a Mix. The SNPs were identified and genotyped within samples producing a raw set of 89,759 biallelic SNPs for four pollen cultivars and a mixed pollen sample (Supplementary Table S3 online). Including the genotyping information of all the pollinated stigmas at all times, we gathered a total of 624,692 biallelic SNPs, of which 30,712 were genotyped and comparable in all samples at 6 h, and 27,767 biallelic SNPs were genotyped and comparable in all samples at 6, 24, and 48 h, showing genetic variability between the treatments (Supplementary Tables S4–S6 online). The total number of heterozygous calls along the genome was higher in the mixed pollen sample, the cross and mix pollinated stigmas compared to other pollen cultivars and pollination treatments at 6 h (Fig. 1). Representative SNP alleles of the RG cultivar and the pollen mix were detected in the PL pollinated stigmas as heterozygous genotype calls. Along the genome several haplotype blocks where found differentiating the apple cultivars genotypes. For example, a 1.16 Mbp long haplotype located, at chromosome eight (Chr 08: 2,109,591–3,276,063 bp), differentiated the pollen apple cultivars and is apparent in the outcross pollinated stigmas as heterozygous calls. The single visit, five visits, self-pollination, and no-pollination control shared the homozygous genotype with the PL pollen sample (Fig. 2). This indicates that RNA-seq based variant genotyping can be used to indicate the presence of non-PL pollen RNA in the stigma and style samples and even to perform parental identification, at least after hand pollination.

**Figure 3:**
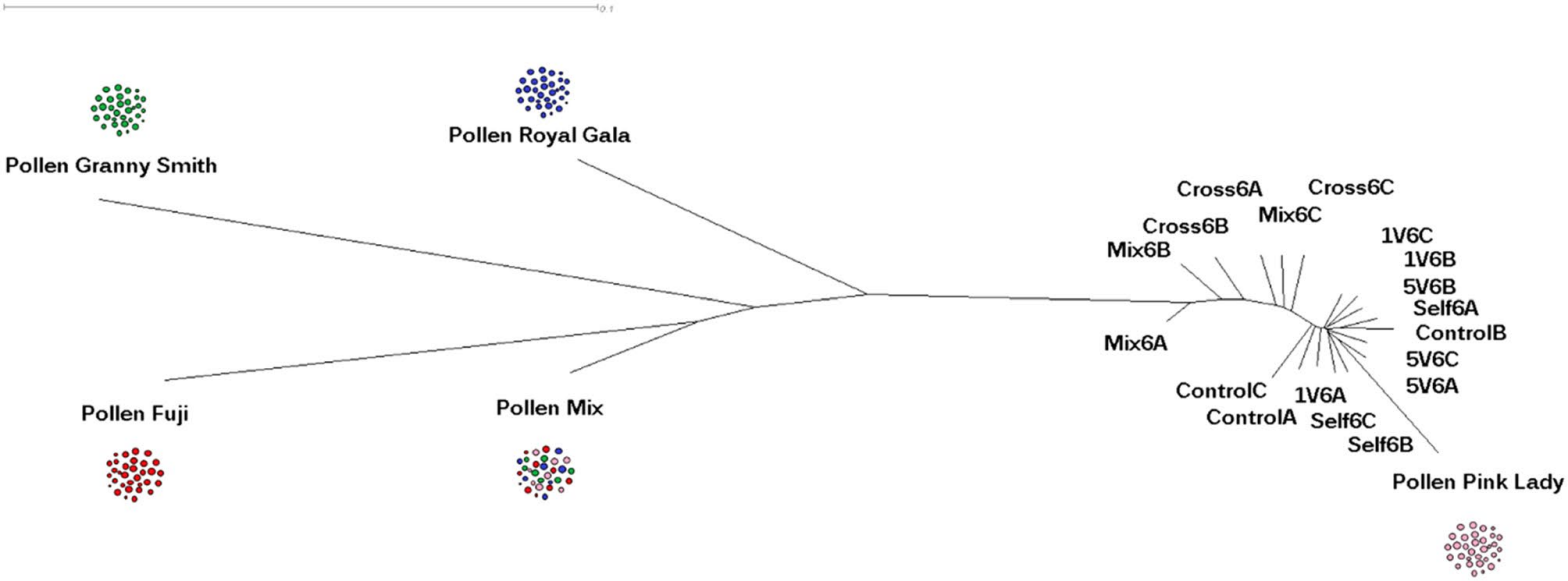
Neighbor-joining clustering analysis of pollination, using the SNPs data set for pollen and pollinated stigmas at 6 h. The neighbor-joining clustering was implemented to graphically examine the genetic similarities. The labels denote honey bee pollination for single (1 V), and five visits (5 V). Bagged flowers correspond to no pollination (control). The hand-pollination treatments self-pollination (Self), cross pollination (Cross), and pollen variety mix pollination (Mix). The genotype calls were obtained after 6 h in triplicates (**A**–**C**) including the pollen cultivars and the pollen mix as references, Pollen Mix; FJ, Fuji (Red); GS, Granny Smith (Green); RG, Royal Gala (Blue); PL, Pink Lady (Pink).

Using the genotyping data, a neighbor-joining clustering analysis was implemented to describe the overall genetic relationship between the different apple pollen cultivars and the pollination treatments at all times (Supplementary Figure S5 online). Using the genotype information at 6 h revealed a distinctive genotype distribution (Fig. 3). As expected, the dendrogram showed two major clusters, representing the pollen samples and the pollinated PL stigmas. In the pollen cluster it is possible to differentiate three cultivars, being GS and FJ pollen the samples more distant genetically compared to the Golden Delicious reference genome. The pollen from RG exhibited higher genetic resemblance to GS pollen. The pollen mix, which comprised three outcross cultivars, were close to the three pure pollen samples. Within a second cluster, the Mix and Cross treatments were most similar to the pollen samples, while the single visits, five visits, self-pollination, and no-pollination samples were tightly clustered, and close to the PL pollen sample. It is interesting to note that the terminal branch length of the PL pollen was greater than those of the pollinated styles, including the Self treatment, suggesting a pollen tissue-specific allele genotype signal.

### Fruit set

Field experiments of fruit set in response to different pollination treatments revealed that the control no-pollination, self-pollination, single honey bee visits, and five honey bee visits were all insufficient for initiating fruit set (Table 2). Of the 360 flowers that were hand pollinated with a mix of non-PL pollen, 227 (63%) achieved initial fruit set and only 130 (36%) progressed until final fruit set. Under field conditions, we found that the outcross mix hand pollination was five times more likely to set fruit compared to open-pollinated flowers and fifteen times more likely to set fruit than flowers that received up to five honey bee visits. Studies have demonstrated that the proportion of flowers that set fruits and seeds increased with the number of visits, after 10 visits 25% of the visited apple flowers reach seed and fruit set success under orchard conditions^33^.

**Table 2:** Initial and final fruit set of open flowers as natural pollination control, bagged flowers for hand-pollination with pollen mix (RG, GS and FJ) and hand self-pollination with Pl pollen.

Treatment	Pollen	n	Initial fruit		Final fruit	
Open flowers		410	92	0.22	58	0.14
Hand pollination	Mix	360	227	0.63	130	0.36
	PL	46	0	0	0	0
No-pollination control		397	4	0.01	2	0.01
1 visit		50	0	0	0	0
5 visits		50	2	0.04	1	0.02

No pollination control, single honey bee visits and five honey bee visits pollination treatments were measured. The initial (30 days) and final fruit set (range 170–249 days) were measured. The relative successful values of fruit set were calculated according to the number of repetitions (n) by each treatment.

## Discussion

Understanding pollen flow by insects is essential to support global food production in pollinator-dependent crops. Here we show that mRNA sequencing and transcriptome analysis were successfully applied to detect pollination events in apple orchards under field conditions. This is relevant as most previous pollination transcriptome analyses have targeted pollen tube gene expression or self-allele recognition mechanisms, in the absence of actual pollinator visits under field conditions. Our approach provides new opportunities to study pollination, and will enable greater capacity to characterize the net fitness direction of plant–insect interactions without the interference of post-pollination processes^4,8,34^.

Pollen tube growth following pollination depends on many factors and the time-course analysis in this study demonstrates that timing is indeed important following a given pollination event^8,35^. The differential gene expression observed at 6 h after cross-pollination presented the greatest number of differentially expressed genes, and a stable signal for pollen genotype detection. In contrast, a lower number of differentially expressed genes were detected at 24, and 48 h after the pollination event, yet a differential expression signal was detected at all pollination treatments nonetheless. Given that the strongest differentiation of gene expression occurred at 6 h in this study, we recommend that future studies focus on the collection of samples at earlier times, starting at 5–20 min for pollen rehydration, pollen activation, pollen tube germination, and early pollen recognition studies. Later times such as 1–6 h will favor the capture of information for pollen tube development, pollen tube competition among cultivar, and pistil response^30,36^. Samples collected long after pollination has occurred, i.e. after two days, could be valuable to identify post-pollination stages to capture wilting, oxidation, allergens, defense response, and fertilisation gene expression^22,29,37–39^.

The mRNA data gathered in this study enabled the identification of genes isoforms expressed in stigmas and styles that were hand-pollinated with RG pollen. The group of genes differentially expressed in this treatment (when compared to the no-pollination control treatment) represent the different molecular functions required for the dynamic process of pollination in wet stigmas^40^. Pollen development, maintenance, vesicle traffic, signaling, pollen tube activation, cytoskeletal dynamic, directional growth, stigma cell wall loosening, calcium gradient, ubiquitination signal, auxin, kinases, allergens, defense response, RNases, and S allele recognition genes were all detected. Likewise, related genes have been identified in maize, apricot, petunias, tomato, tea, tobacco, brassica, pear, *Arabidopsis thaliana*, almonds, and orchids pollination transcriptome analyses^19–23,25,29,38,41–44^.

By combining the genotyping data, the differential expression analysis, and the traditional fruit set experiments, it was possible to evaluate the pollination effectiveness of single and five honey bee visits, under field conditions in real commercial orchards. This approach demonstrates that effective cross-pollination is not achievable with single or five honey bee visits, under the tested orchard conditions. To our knowledge, this is the first time that pollen–stigma-style mRNA expression analysis has been conducted after a pollinator visit to any plant. The gene isoform expression analysis only presented a few genes down and up-regulated in the honey bee visits treatments with an increase towards downregulation at 48 h. In addition, the genotyping matrix differentiated the cross and mix-pollinated stigmas from those that were visited by honey bees. The heterozygous call rate and the clustering analysis showed a similarity between the single and five visit pollination treatments, the self-pollination, and the no-pollination control. Field data in the same orchards indicate that up to five honey bee visits are not enough to result in a successful outcross-pollination event^33^. This highlights the importance of increasing the number of visits above ten, to capture the natural outcross pollination gene expression profile. Further, our results indicate that it is possible to characterize the sequence of pollination events, during the entire life span of a mature flower. Nonetheless, given the differences in cross-compared to self-pollinated treatments, the method is likely suitable for further investigation of detection limits due to low outcross conspecific pollination events or the presence of heterospecific pollen as a source of interference^45,46^. When apple flowers in the field were hand-pollinated with a single cross cultivar, pollen tube gene expression was upregulated, outcross pollen was detectable through total heterozygous calls and cultivar specific SNPs markers.

In this study, pollen mRNA reads were used to capture SNP markers that differentiate pollen from four apple cultivars, the presence of multiples alleles in a mixed pollen sample, and outcrossed stigma and style tissue samples. This allowed us to distinguish cultivar outcross pollination events from self-pollination or no pollination, which has not yet been possible with traditional barcoding markers. Since the first application of DNA barcoding to pollen, the field of pollination ecology has now expanded its methodological toolbox of DNA sequencing for taxonomic identification and many other palynology applications^13,16^, including a greater understanding of pollinator diet preferences^14,15,17,47–49^. Within crop systems, however, a different molecular approach is required if a taxonomic resolution is needed at the cultivar level. Traditionally, mRNA sequencing data has been used only to study differential gene expression, although plant transcriptome sequencing has also made significant contributions to phylogenomics, ecological genetics, genome evolution, domestication, and improvement^50–53^. The results of this study indicate it is indeed possible to use other standard high throughput genotyping methodologies, such as whole-genome DNA sequencing short or long reads, reduced representation sequencing (e.g. RAD-seq) or SNP arrays, to genotype pollen grains to the cultivar-level to understand crop pollen flow, pollen–stigma interactions, pollinator effectiveness, and pollinator foraging behavior.

## Conclusion

Next-generation sequencing technologies can be used to study pollination services and insect pollinator effectiveness under real commercial orchard conditions. Here we demonstrated the capacity of RNA-seq to (1) detect cross-cultivar pollination events using post-pollination gene expression and (2) act as a precise genotyping tool for donor identification. We detected over four hundred upregulated genes isoforms when PL stigmas were hand-pollinated with RG pollen. Pollen tube specific genes were revealed as expression markers involved in the pollen tube activity through the stigma and style tissue. The genotyping matrix allowed corroboration between the cross cultivar pollination events by the allele recognition of the different pollen cultivars and the heterozygous state of the outcross cultivar pollen–stigma-style samples. This is a promising approach for identifying pollen donor cultivars in the field.

## Materials and methods

We conducted observations of insect visitors to Pink Lady (PL) apple flowers (*Malus domestica* var. Cripps Pink-Pink Lady) during the flowering season of September–October in both 2017 and 2018. Fruit set observations were carried out in 8 apple orchards with multiple cultivars, located in Stanthorpe, Queensland, Australia (Supplementary Table S7). Apple is an ideal crop to investigate pollination at the molecular level, given its need for cross-pollination from different compatible cultivars for optimal fruit set and quality^54–57^. Royal Gala (RG) and PL are the two most commonly grown apple cultivars in this region. In each orchard, apple trees were arranged in rows approximately 4.5 m apart, with trees planted approximately every 1.25 m within rows. Apple cultivars typically alternate between rows, or in some cases, blocks of trees of the same cultivar are grouped and planted adjacent to another. Granny Smith (GS), and Fuji (FJ) and RG were growing in the area and were used as pollen donors in addition to PL.

**Figure 4:**
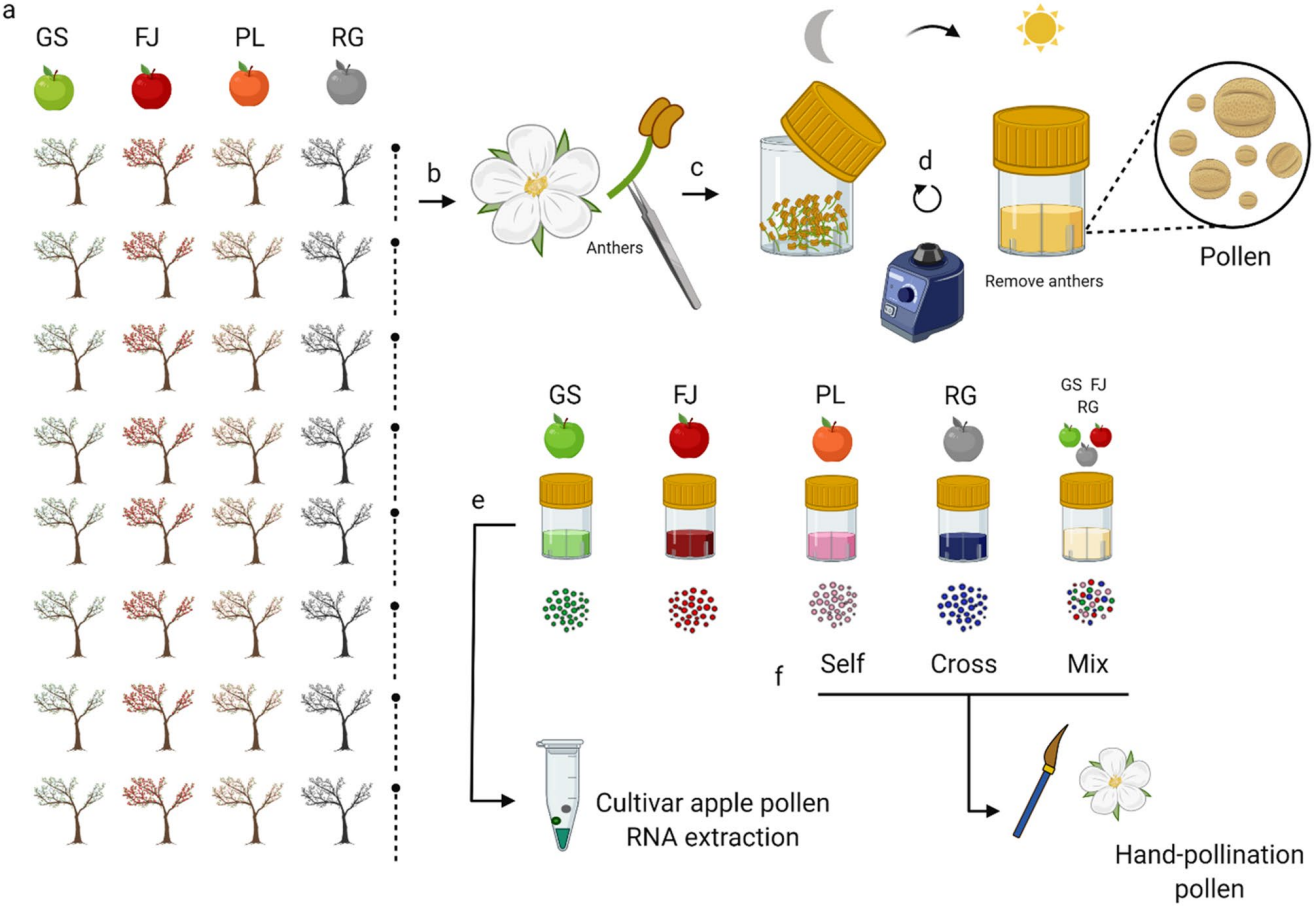
Methodology for pollen variety collection. (**a**,**b**) Most farms have different cultivars in rows. We collected the anthers of each young flower from each cultivar in a separate sterile container. (**c**,**d**) The anthers were dried overnight and vortexed to liberate the pollen grains. The next day the anthers were removed with sterile tweezers leaving only pollen grains in the container. The different apple pollen cultivars were collected and a 1:1:1: ratio pollen mix sample was composed using RG, GS, and FJ. (**e**) The collected pollen was used for RNA extraction and posterior mRNA sequencing as pollen cultivar library references. (**f**) Pollen collected from PL was used for the hand self-pollination treatment, RG pollen for cross-pollination treatment and the composed mix mix-pollination treatment. (Schematic created with BioRender.com).

The anthers of 200 flowers from 20 trees of each row of the four cultivars were collected into 75 ml individual sterile vials. The anthers were dried overnight and dehisced with a vortex. The anthers were then removed from the tube, leaving only pollen grains from the different cultivars. These samples were included in sequencing as a pollen library reference for cultivar identification (Fig. 4). The same methodology was used to collect pollen for the hand-pollination treatments.

**Figure 5:**
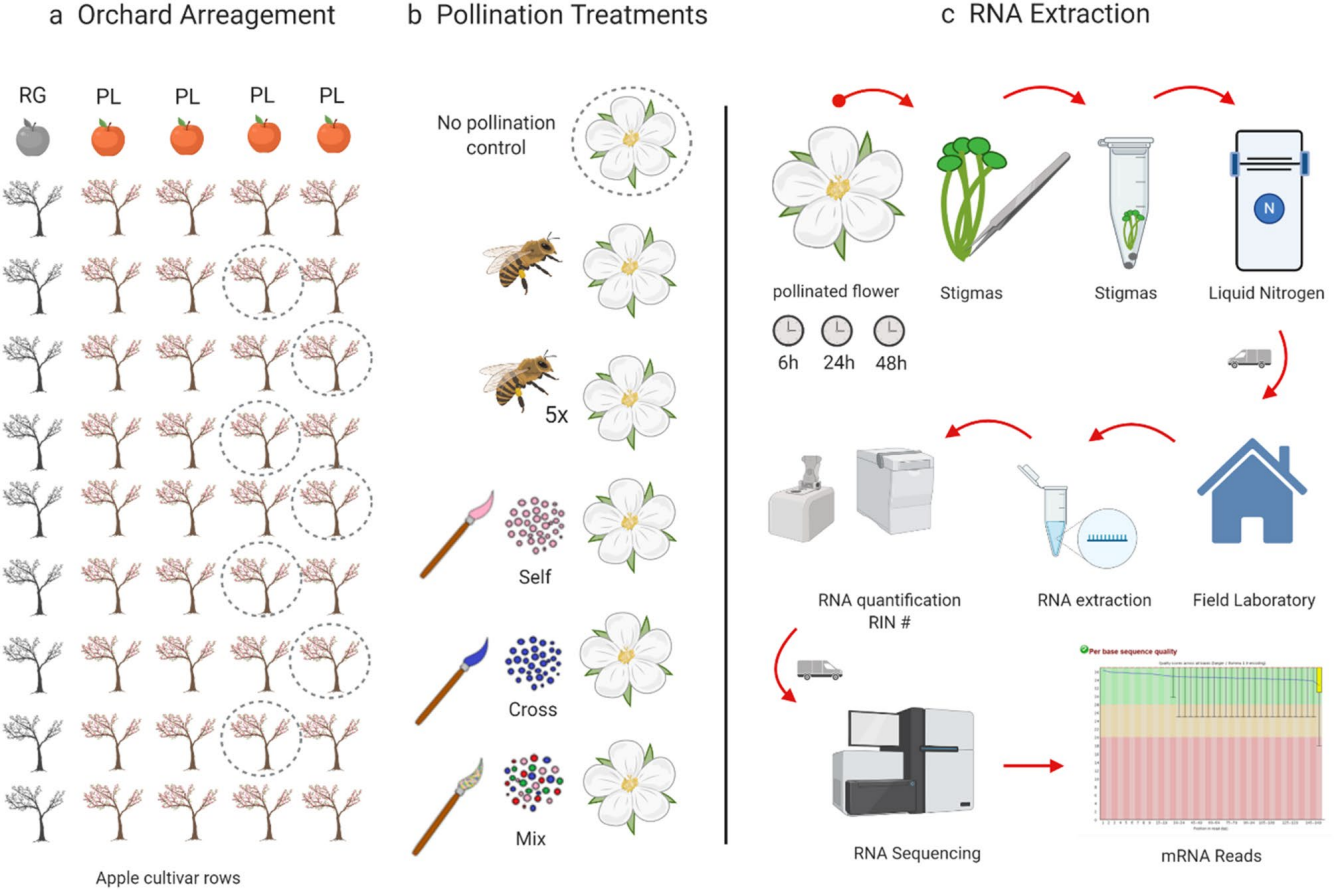
Methods for pollination treatments. (**a**) Cultivar orchards arrangements and tree selection for pollination treatments. The branches with flower buds were selected, tagged, and bagged to avoid pollinator’s visits. (**b**) When flowers were mature the bags were opened and the different pollination treatments were conducted. Bagged flowers correspond to no pollination (control), natural pollination (honey bee), for single visit (1 V), and five visits (5 V). Hand-pollination treatments (brush) were self-pollination, cross pollination, and pollen variety mix pollination. (**c**) The RNA extraction was conducted at three different times i.e. 6, 24 and 48 h post-pollination. The flower stigmas were collected and placed in 1.5 ml microcentrifugue tubes for immediate immersion in liquid nitrogen. The samples were carried to a field laboratory for RNA extraction and quantification. The samples were transported for mRNA sequencing at Ramaciotti Centre, Sydney, Australia. (Schematic created with BioRender.com).

A total of 300 PL flower buds from 30 trees were selected from two rows of 70 PL trees located 10 m away from the nearest RG row. Each bud was enclosed in individual organza bags to exclude insect visits. Each flower was then divided into one of six treatments (Fig. 5).

The six treatments were:

1. No-pollination (control): bagged mature flowers were sampled without applying any external pollen to the flower or pollinator visit.
2. Single honey bee visit (1 V): bagged mature flowers were unbagged and then observed until they were visited once by a honey bee, then rebagged until processing.
3. Five honey bee visits (5 V): bagged mature flowers were unbagged and then observed until they were visited five times by honey bees, then rebagged until processing.
4. Self-pollination (self): bagged mature flowers were hand-pollinated using a paintbrush with Pink Lady pollen on Pink Lady stigmas
5. Cross-pollination (cross): bagged mature flowers were hand-pollinated using a paintbrush with Royal Gala Pollen, the pollen donor.
6. Mixed-pollination (mix): bagged mature flowers were hand-pollinated using a paintbrush with a 1:1:1 mixture of pollen from three other apple cultivars: RG, GS, and FJ.

### RNA extraction and sequencing

RNA was extracted from five stigmas of five flowers for each of the different treatments. Each of the different treatments was sampled at three different time intervals, i.e. 6, 24, and 48 h after the pollination event. To obtain the best possible RNA, the RNA extraction was conducted in the field. The stigmas were removed from the flowers using sterile stainless steel tweezers and rapidly enclosed in sterile 1.5 ml Eppendorf Safe-lock tubes (Germany), then immediately placed in liquid nitrogen. The samples were promptly taken to the field laboratory, and the frozen tissues were pulverized using a bead rotor (Biospec/Cole Parmer Mini-beat beater) before the extraction using Qiagen RNA/sRNA/DNA extraction kit accordingly to the manufacturer’s instructions. RNA was also extracted from pollen grains of the four cultivars and the pollen mixture. The extracted total RNA was quantified with a NanoDrop 8000 by measuring absorbance at 260 nm and 280 nm (A2560/A280) and electrophoresis gel with a 2100 Bioanalyzer Agilent technologies to measure the concentration and integrity. The best three replicas in terms of their concentration and quality by RNA Integrity number were selected. In total forty-nine of the pollination treatment stigmas, four pollen samples of each cultivar, and a mixed sample containing all pollen cultivars in equal proportions (FJ, GS, and RG) were sequenced. The mRNA was sequenced using a NovoSeq Illumina 6000, for 150 bp long paired ended reads, S1 flowcell type. Sequencing and mRNA library construction were conducted by an external provider (Ramaciotti Centre, Sydney, Australia).

### RNA mapping, differential expression analysis and genotyping

The mRNA reads sequenced adaptors were removed with Trimmomatic-v0.32^58^. The quality of the reads was measured using FastQC^58,59^. The FastQC report showed read quality above Q20 in all our samples and, only Q18 scores at the last 3 bp of the reads. We tested no trimming, trimming 1, 3, 5, and 10 bp. Trimming 3 bp exhibited the best relation of increased mapping percentage without compromising total read length. The reads were mapped to the indexed latest reference Golden Delicious apple genome^60^. Using HISAT2-2.1.0, with − 3/– trim3 (3) and − 5/− trim5 (3) parameter the reads were aligned ignoring the first and the last three bp^61,62^. This approach was taken because sequencing data can be prone to error at both ends of the sequenced fragment and, it is common to find the base quality reduced at the ends of the reads in RNA-seq experiments due to the variable sequence nature of splicing sites carried on by mRNAs^63,64^. The mapped reads were then assembled using Stringtie-1.3.5 integrating information on known splice sites index and the transcriptome annotation available with the reference genome^62,65^. A metagenomics analysis with kaiju (a fast and sensitive taxonomic classification for metagenomics webserver tool) was implemented for a 2.5 Million reads subset to identify the microbes associated with an environmental sample^66^. For differential expression analysis, the transcriptome assemblies of each treatment and their predicted abundances were compared with the R package “Ballgown”^67^. Fragments per million kilobases (FPKM) was used as a measure of expression to compare all different pollination samples at all times against the no-pollination control. Differentially expressed genes (p value bellow 0.05) were called in conjunction with false discovery rate test (q value below 0.05). A four-fold change was observed in the FPKM distribution between compared groups of samples and the differentially expressed gene list was obtained^62,65^.

The pollen cultivars were genotyped using the NGSEP-4.0.1 pipeline^68^. Variants were identified and genotyped running the modules “*FindVariants, MergeVariants and MergeVCF*” over multiple samples as specified in the user manual. For a conservative RNA genotyping approach we targeted only the central region of the mRNA sequences. The reads were mapped taking into account the splicing sites recognized by Stringtie and then evaluated avoiding ten bp at each end of the reads. The pollen was genotyped, and the pollination events sampled with a minimum quality score of Q40. The module “VCFAnnotate” was run to generate a VCF file including the functional information related to each variant, based on the GFF reference annotation file. The variant annotation brings information about the SNPs in relation to the gene annotation. To avoid missing data, the “VCFFilter” module was implemented to select only the genomic locations where all pollen samples and pollinated stigmas could be compared. The general statistical parameters including the total heterozygous call of the resulting VCF file were calculated with the “VCFSummaryStats” module. Although mixtures of alleles in RNA from pollinated stigmas are not well characterized by diploid genotypes, we used apparent heterozygous genotype call state as a conservative indicator of the presence of multiple alleles in the cultivar pollen mix samples and non-self-alleles in the PL stigmas. An identity-by-state distance measure for each pair of samples was calculated, averaging differences in genotype calls across the identified SNPs. The genotype classification was calculated using the “DistanceMatrixCalculator” module. The VCF results and the distance matrix were transformed with the “VCFConverter” module for visualization with Flap-Jack and SplitsTree^68–71^.

### Field data on fruit set as a result of pollination treatments

To quantify fruit set in relation to the same pollination treatments used in RNA-seq analyses, 1313 flowers from 8 orchards were selected to measure fruit set. Trees and flowers used in the fruit set study were different from those used for molecular testing to avoid impacts on fruit set related to the removal of styles before fruit development. Each orchard had a different cultivar arrangement and differing proportions of cultivars (Supplementary Table S6 online). Donor flowers were collected from co-flowering apple cultivars present within the corresponding orchard block. The anthers were removed from their filaments with fine forceps and left in an open vial overnight at room temperature (14–25 °C). A paintbrush was used to deposit pollen onto the stigmas of recipient flowers. The fruit set was recorded as the proportion of fruits produced by treated flowers. The proportion of initial fruit set was recorded approximately 30 days after treatment and at harvest time (mean age at harvest: 192 ± 15 days, range 170–249 days) to compare early fruit set with final fruit set and as a result of any fruit thinning by growers. We initially collected stigmas for gene expression from flowers visited by honey bees once, three times, and five times. However, when we obtained fruit set results, we discovered that five visits were insufficient for fruit set. We therefore conducted an additional field season to determine what number of visits was required for optimal fruit set. We tagged these higher visitation flower samples to measure fruit set and demonstrate that a greater number of visits (> 10) was required to result in apple fruit production. Further, on some days honey bee visitation rates were low and waiting in the field for 10+ visits on a single flower took most of the day. Hence, we were also concerned that waiting for additional flower visits could result in variations in flower age that in turn, could impact gene expression. Additional future studies are required to measure gene expression with higher numbers of visits and possibly the entire duration in which a flower is mature.

## Supplementary Information

Supplementary Information 1.

Supplementary Information 2.

Supplementary Information 3.

Supplementary Information 4.

Supplementary Information 5.

Supplementary Information 6.

Supplementary Information 7.

Supplementary Information 8.

## Data Availability

All reads generated by Illumina sequencing have been deposited in the Sequence Read Archive (SRA) database (http://www.ncbi.nlm.nih.gov/sra) under BioProject PRJNA657757.
