# Supplementary material for: Using RNA-seq to characterize pollen–stigma interactions for pollination studies
Source: Sci Rep. 2021 Mar 23;11:6635. doi: 10.1038/s41598-021-85887-y (PMC7988043; doi:10.1038/s41598-021-85887-y)
Supplement: Supplementary file 8 — Supplementary Information 8. [file 41598_2021_85887_MOESM8_ESM.pdf]

Supplemental information tables, figures, and scripts for
Using RNA-seq to characterize pollen-stigma interactions for pollination studies

Juan Lobaton[1,3], Rose Andrew[1], Jorge Duitama[2] , Lindsey Kirkland[1], , Sarina Macfadyen[3], Romina Rader[1]

[1]School of Environmental and Rural Science, University of New England, Armidale, Australia; [2]Systems and Computing, Engineering Department, Universidad de los Andes, Bogota, Colombia; [3]CSIRO, Clunies Ross St. Acton, ACT, Australia.

Corresponding author

Juan Lobaton, Email jdlgarces@gmail.com

Supplementary Table S1: Sequencing statistics per library. Total number or reads, total number of bases, GC and content by sample, mapping percentage to the reference genome, and aligning percentages to other species in the NCBI data base by running the SRA submission.

Supplementary Table S2: List of genes isoforms differentially expressed by four-fold change at 6, 24, and 48 hours, by pollination treatment. Apple gene id, gene description, and expression estate down-regulation up-regulation (separated Excel file).

Supplementary Table S3: SNPs calls results for pollen cultivars. Overall statistics of the SNPs markers genotyped in pollen cultivars Fuji, Granny Smith, Royal Gala, Pink Lady and a Mix (separated Excel file).

Supplementary Table S4: Dataset variants call format file (VCF) of pollen and pollination treatments at 6 hours. Information of the SNPs markers location in the apple genome for the genotyped SNPs in the pollen samples and the pollinated stigmas at 6 hours after pollination (separated Excel file)

Supplementary Table S5: SNPs calls results for pollen and pollination treatments at 6 hours. Overall statistics of the SNPs markers genotyped in the pollen samples and the pollinated stigmas at 6 hours after pollination (separated Excel file).

Supplementary Table S6: SNPs calls results pollen and pollination treatments at 6, 24, and 48 hours. Overall statistics of the SNPs markers genotyped in the pollen samples and the pollinated stigmas at 6, 24 and 48 hours after pollination (separated Excel file).

Supplementary Table S7: Orchards cultivar arrangement. List of orchards and apple cultivars arrangements used in this study (separated Excel file).

Supplementary Figure S1. Total reads and mapping percentage. Total mRNA reads per sample (Grey bars) and mapping percentage of reads aligned to the reference genome *Golden delicious* of pollination and pollen samples (Yellow line).

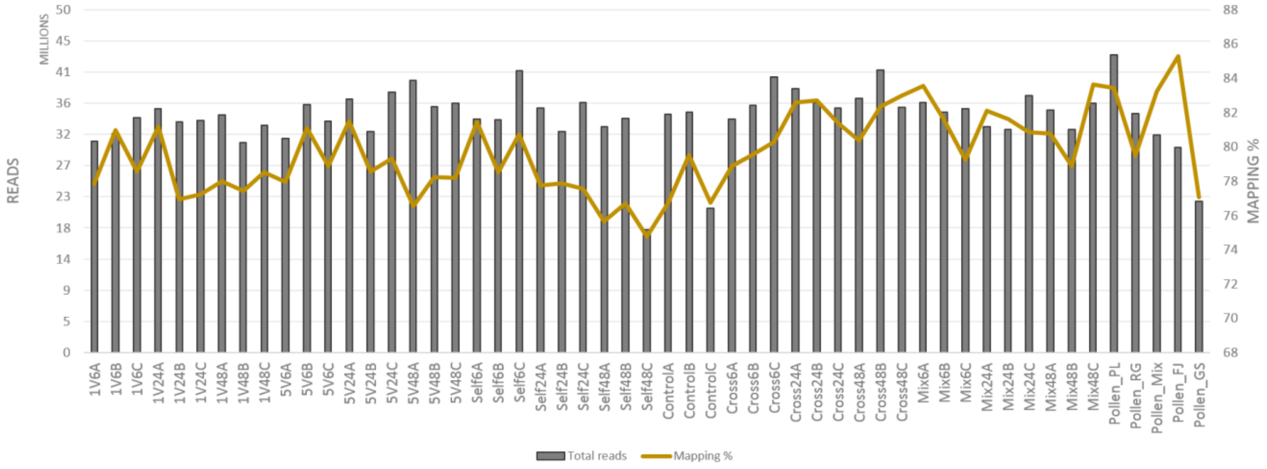

Supplementary Figure S2. Summary of all pollination treatments comparison at three times 6, 24, and 48 hours. Differential gene expression results, p-value 0.1 (Blue), false discovery rate q-value 0.05 (Green), and four-fold change (Yellow).

	6h					24h					48h				
**p-value**	Mix	Self	5 Visits	1 Visits	Control	Mix	Self	5 Visits	1 Visits	Control	Mix	Self	5 Visits	1 Visits	Control
Cross RG	1250	5060	3835	4970	4133	1833	914	9301	4647	1876	1422	4548	4210	7500	4028
Mix		4891	3375	3219	2438		1833	7830	4686	1646		8179	6089	8642	5624
Self			3018	3675	2184			4092	1847	1581			7312	2486	3364
5V6				4019	2900				1889	3052				7736	3843
1V6					2567					4327					4327
**q-value**															
Cross RG	0	0	0	4	0	0	0	0	0	0	0	0	0	4	0
Mix		0	0	0	0		1	0	0	0		6	0	8	0
Self			0	0	0			0	0	0			24	0	0
5V6				0	0				0	0				2	0
1V6					1					0					0
**Fold change**															
Cross RG	0	5	3	1	429	0	0	3	342	36	0	2	0	0	12
Mix		0	0	0	1		2	0	3	2		5	0	6	24
Self			0	0	0			35	373	28			0	0	85
5V6				0	1				0	1				1	19
1V6					0					0					6

10000 / 1000; 30 / 0; 450 / 0

Supplementary Figure S3. Transcriptomic gene isoform analysis of pollination. The transcriptome result of the stigmas pollinated by honey bees and hand pollinated were evaluated against the No pollination control. a. Results of the log2 Fragment Per Kilobase Million (FPKM) distribution of pollination treatments (y axis), compared to the no pollination control (x axis) at 6, 24, and 48 hours. The red circles represent the four-fold gene changes in expression by treatments. b. Gene results distribution to q-value test and four-fold change test (green). The red lines indicate two-fold changes in expression. The results present the overall gene expression regulation direction of the pollination treatments over time.

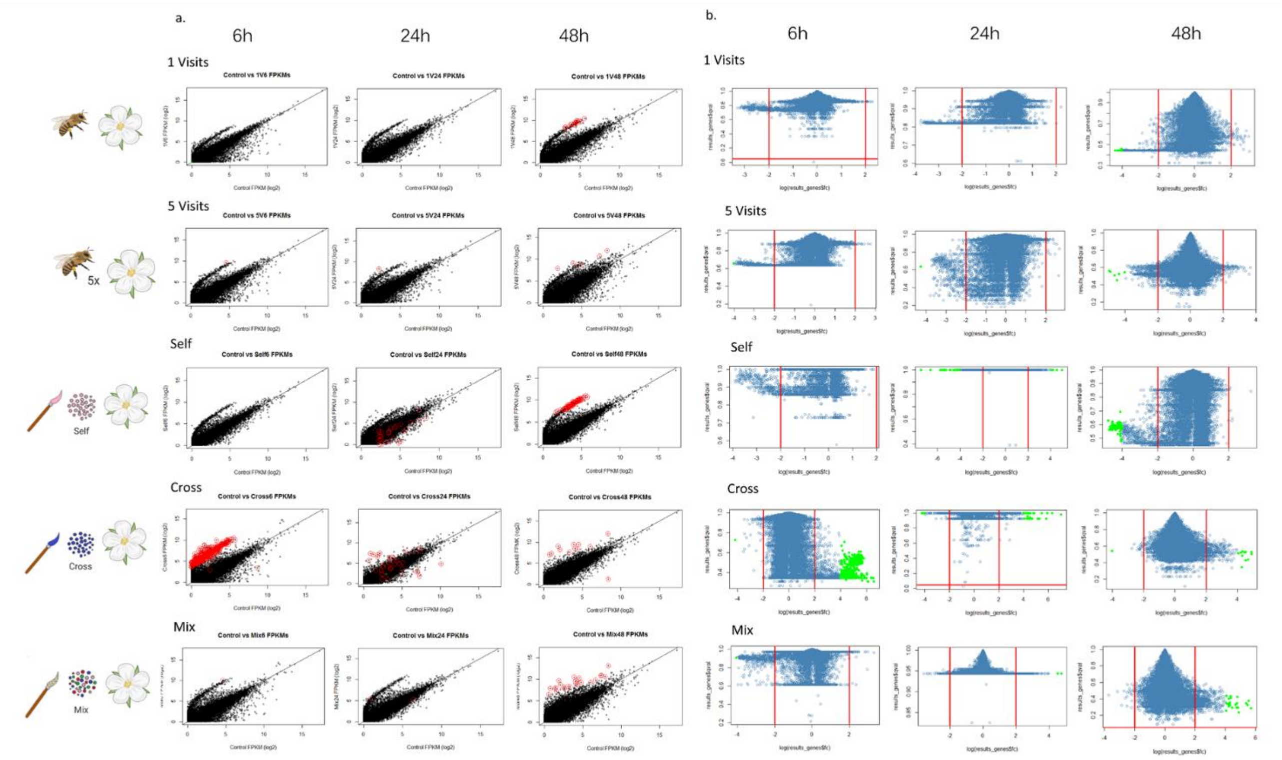

Supplementary Figure S4. Heat map representing the gene expression results of all samples compared to the no pollination control. The genes were differentially expressed in the cross 6 hours and self-pollination 48 hours. The gene expression matrix showed important pollen tube genes differentially expressed on the cross pollinated treatments especially at 6 hours compare to the no pollination control.

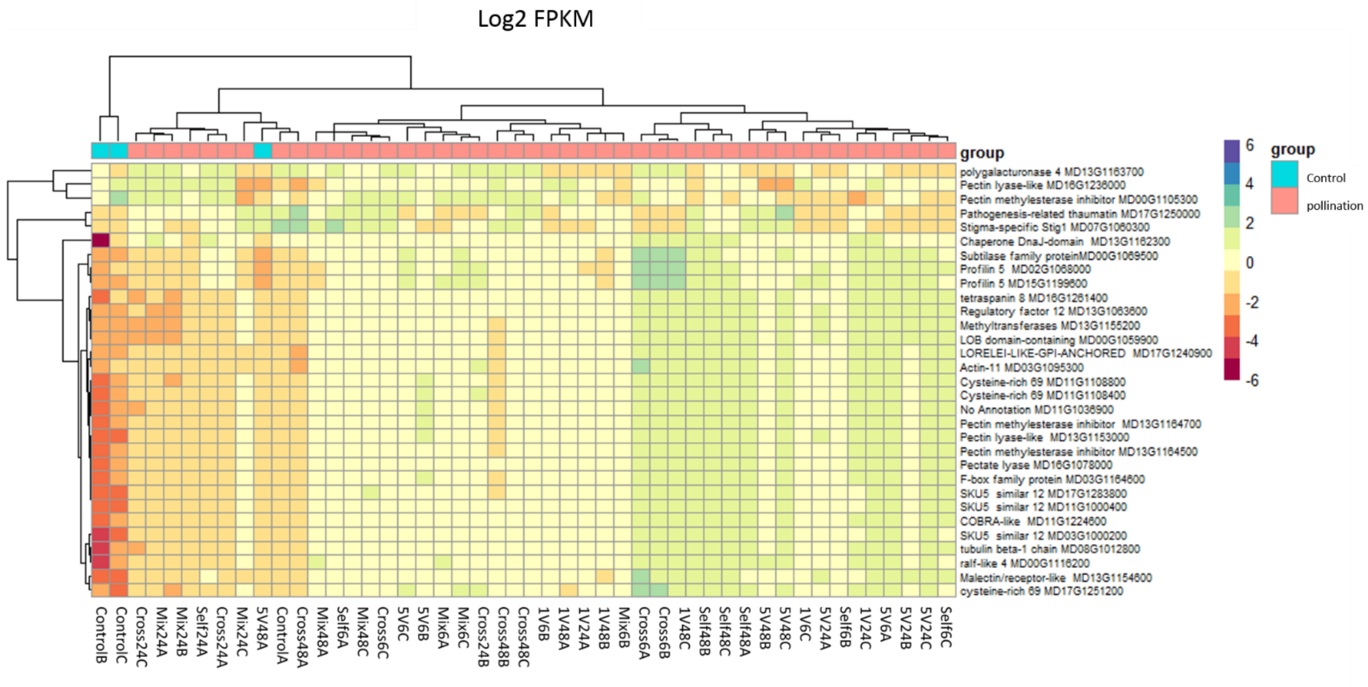

Supplementary Figure S5. Neighbor-Joining dendrogram using the pollen and pollinated samples at all times (n=52). See three different zooms to detect 6 hours outcross pollinated stigmas, 24 and 48 hours outcross pollinated stigmas, and the no-pollinated control with 1 visits, 5 visits, and self-pollination stigmas at all times.

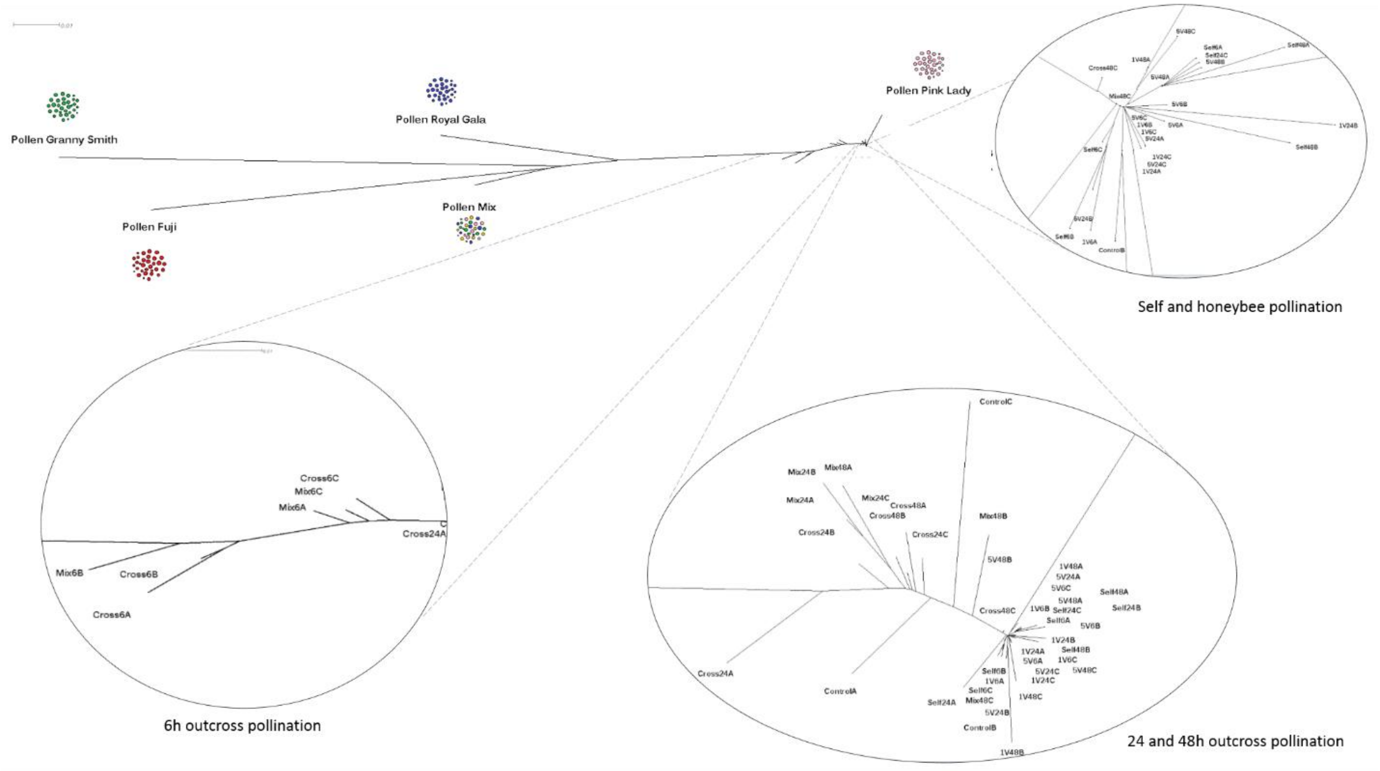

Supplemental Scripts. Scripts software's and command line used to analysed RNA-seq to characterize pollen-stigma interactions for pollination studies

Supplemental script 1: Check reads quality with FastaQC

```
## Reads validation and quality view

# FastaQC

zcat *fastq.gz | fastqc stdin
```

Supplemental script 2: Built reference genome indexes with Hisat2

```
## indexing reference genome

#HISAT2

wget ftp://ftp.ccb.jhu.edu/pub/infphilo/hisat2/downloads/hisat2-2.1.0-Linux_x86_64.zip

unzip hisat2-2.1.0-Linux_x86_64.zip

cd hisat2-2.1.0

./hisat2

# make splice sites reference index

qsub -I /Bin/hisat2-2.1.0/extract_splice_sites.py /Reference.gtf > Reference.ss

# make exon index

extract_exons.py /Reference.gtf > Reference.exon

# Build nuclear reference sequence index

/bin/hisat2-2.1.0/hisat2-build Reference.fa Reference.idx

# Build mitochondria index

/bin/hisat2-2.1.0/hisat2-build Malus_domestica_mitochondria.fa
Malus_domestica_mitochondria

# Build chloroplast index

/bin/hisat2-2.1.0/hisat2-build Malus_x_domestica.v1.0.chloroplast.fa
Malus_x_domestica.v1.0.chloroplast.
```

Supplemental script 3: Mapping the reads to a reference genome with Hisat2

```
## Mapping reads to reference genome trimming 3 bp at each end

hisat2 --dta -5 3 -3 3 -q -x /reference -1 /Reads_R1.fastq.gz -2 /Reads_R2.fastq.gz -
S Reads.sam

## mapping using the splice sites information and trimming 3 bp at each end

hisat2 --dta -5 3 -3 3 --known-splicesite-infile /References.txt -q -x /References.idx
-1 /Reads_R1.fastq.gz -2 /Reads_R2.fastq.gz -S reads.sam

## Conver SAM to binary BAM
```

```
samtools/1.9.0
samtools sort -o Reads.bam Reads.sam
## Check overall mapping .bam files
grep overall *.log
---------------------------------------
---------------------------------------
```

Supplemental script 4: Transcriptome analysis with StringTie

```
## reads transcript assembler
# StringTie
tar xvfz stringtie-1.3.5.Linux_x86_64.tar.gz &
make release
tar xvf stringtie-1.3.5.tar.gz &
stringtie <aligned_reads.bam> [options]*
stringtie /Reads.bam -o Reads_alignments.gtf &
## Genome tools gene annotation (gff) index
wget genometools-0.6.5.tar.gz
tar xvf genometools-0.6.5.tar.gz
make
gt -gff3 -retainids -addintrons input_gff3 > output_gff3
-------------------------------------
-------------------------------------
```

Supplemental script 5: Expression levels with StringTie

```
## RNA expression levels
# StringTie merge list of trnascript assemblies
stringtie --merge -o strigtie_Merge.gtf MergelistGTF.txt
# measure expresssion levels counts control
stringtie -e -B -G strigtie_Merge_test.gtf -o Reads_Exp.gtf ../Reads.bam
```

measure expression levels incorporating splicing sites

stringtie -e -B -A -C -G reference.gff3 -o Reads_exp.gtf ../Reads.bam &

data analysis and visualization with ballgown in Rstudio

Ballgown_input_files: count files (*ctab files) for each sample

e_data.ctab: exon-level expression measurements.

i_data.ctab: intron- (i.e., junction-) level expression measurements

t_data.ctab: transcript-level expression measurements

e2t.ctab: table with two columns, e_id and t_id, denoting which exons belong to which transcripts

i2t.ctab: table with two columns, i_id and t_id, denoting which introns belong to which transcripts

----

Supplemental script 6: Differential expression analysis with Ballgown

Rstudio enviroment

library(ballgown)

setwd("/Workspace")

data_directory = system.file('samples', package='ballgown')

bg = ballgown(dataDir='Samples', samplePattern='Sp', meas='all')

Print sample names

sampleNames(bg)

Create a dataframe with sample names and their group (normal and tumor) and append this data as phenotypic data of samples

pData(bg)=data.frame(id=sampleNames(bg), group=rep(c("control", "control", "control", "treatment", "treatment", "treatment")))

Look at the metadata of samples

pData(bg)

Extract Transcript level expression measurements. We can extract all the information

```
# (FPKM and Coverage) or individual (FPKM or Coverage). Let us extract all
information.

tx_table = texpr(bg,  "all")

# Extract Transcript level expression measurements. To extract FPKM

fpkm = texpr(bg,meas="FPKM")

# Calculate the differentially expressed transcripts using group information (control vs
treatment) and FPKM information.

det = stattest(bg, feature="transcript", covariate="group", getFC=TRUE, meas="FPKM")

# Calculate differentially expressed genes instead of transcripts,

deg = stattest(bg, feature="gene", covariate="group", getFC=TRUE, meas="FPKM")

results_txns = stattest(bg, feature='transcript', getFC = T,
covariate='group',meas='FPKM' )

# Calculate the differentially expressed transcripts using group information (control vs
treatment) and FPKM information.

# make the ballgown object:

##Extract expression (expr) values (as FPKM) for transcripts (t)

transcript_fpkm = texpr(bg, 'FPKM')

##Extract expression (expr) values (as coverage) for transcripts (t)

transcript_cov = texpr(bg, 'cov')

##Extract expression (expr) values (as FPKM, Coverage) for transcripts (t)

whole_tx_table = texpr(bg, 'all')

##Extract expression (expr) values (as FPKM, Coverage) for junctions and introns (t)

whole_intron_table = iexpr(bg, 'all')

##Extract expression (expr) values (as FPKM) for exons (e)

exon_mcov = eexpr(bg, 'mcov')

##Extract expression (expr) values (as FPKM) for junction reads (i)

junction_rcount = iexpr(bg)

##Extract expression (expr) values (as FPKM) for genes (g).

gene_expression = gexpr(bg)
```

```
##Look at the exon, intron and transcript data

structure(bg)$exon

structure(bg)$intron

structure(bg)$trans

## Store mapping between exon and transcripts

exon_transcript_table = indexes(bg)$e2t

## Store mapping between transcripts and genes

transcript_gene_table = indexes(bg)$t2g

# how many transcripts are there

length(rownames(transcript_gene_table))

# how many genes are there

length(unique(transcript_gene_table[,"g_id"])) #Unique Gene count

------------------------------------------

------------------------------------------

## transcript stats

## plot average transcript length

hist(whole_tx_table$length, breaks=50, xlab="Transcript length (bp)",
main="Distribution of transcript lengths", col="steelblue")

# how many transcripts are there per gene? count the number of genes and count the
number of transcripts pere gene and plot it.

counts=table(transcript_gene_table[,"g_id"])

## extract gene names and transcript names

gene_names=data.frame(SYMBOL=unique(rownames(gene_expression)))

## create sample meta data frame

phenotype_table= data.frame(id=sampleNames(bg), group=rep(c("control", "control",
"control", "treatment", "treatment", "treatment")))

pData(bg) =phenotype_table

## differential transcript expression time
```

results_txns = stattest(bg, feature='transcript', getFC = T, covariate='group',meas='FPKM')

Extract transcript names

t.ids=whole_tx_table[,c(1,6)]

Unique transcript names and ids

t_names=unique(whole_tx_table[,c(1,6)])

merge transcript results with transcript names

results_txns_merged = merge(results_txns,t.ids,by.x=c("id"),by.y=c("t_id"))

head(results_txns_merged)

Calculate differentially expressed genes and use FPKM in calculating # # # # differential gene expression

results_genes = stattest(bg, feature="gene", covariate="group", getFC=TRUE, meas="FPKM")

boxplot(gene_expression, col=rainbow(6), las=2, ylab="log2(FPKM)", main="FPKMs 1V24 vs Cross24")

boxplot(log2(gene_expression+ 1), col=rainbow(6), las=2, ylab="log2(FPKM)", main="log transformed distribution of FPKMs for all 6 samples")

FPKM values are not logged. Hence fold change (FC) is not also logged. Log

fold changes and store it in logfc

logfc = log2(results_genes[,"fc"])

columnresults_genes = logfc

Identify the genes (rows) with adjusted p-value (i.eq-value) < 0.05

qsig=which(results_genes$qval<0.05)

correlation plot between Average expression of control samples Vs average expression of treatment samples

Convert the matrix to data

gene_expression=as.data.frame(gene_expression)

create normal means column

```
gene_expression$Sp_controls=rowMeans(gene_expression[,c(1:3)])

# create tumor means column

gene_expression$Sp_treatments=rowMeans(gene_expression[,c(4:6)])

# to avoid log 0, add 1 to log values. FPKM values are not normalized

x=log2(gene_expression[,"Sp_controls"]+ 1)

y=log2(gene_expression[,"Sp_treatments"]+ 1)

plot(x=x, y=y, pch=1, cex=0.5, xlab="controls FPKM (log2)", ylab="treatments FPKM (log2)", main="controls vs treatments FPKMs")

abline(a=0, b=1)


# Add statistically significant genes to the plot in green color

xqsig=x[qsig]

yqsig=y[qsig]

points(x=xqsig, y=yqsig, col="green", pch=1, cex=0.8)

## upregulated by 16 fold and downregulated by 16 fold.

str(results_genes$fc)

fsig=which(abs(log(results_genes$fc))>4)

xfsig=x[fsig]

yfsig=y[fsig]

points(x=xfsig, y=yfsig, col="red", pch=1, cex=2)

## legend

legend_text = c("Significant by Q value", "Significant by Fold change")

legend("topright", legend_text,bty="n",pch = c(19,19), col=c("green","red"))

# label the significant genes

install.packages("calibrate")

library(calibrate)

textxy(xfsig,yfsig, cex= 0.5, labs=row.names(gene_expression[fsig,]))

## fold change
```

gene_names_foldchange=row.names(gene_expression[fsig,])

write.table(gene_names_foldchange, "/Workspace/Sig_gene_expression_Foldchange_controls_tratments.txt", sep="/t")

gene_names_qvalue=row.names(gene_expression[qsig,])

write.table(gene_names_qvalue, "/Workspace/Sig_gene_expression_Qvalue_controls_treatments.txt", sep="/t")

Volcano plot

Filter genes by log fold change by 16 fold

fc_sig_results_genes=which(abs(log(results_genes$fc))>4)

Extract genes with fold change by 4 fold

fc_sig_results_genes_plot=results_genes[fc_sig_results_genes,]

plot

plot(log(results_genes$fc),results_genes$qval, col="steelblue", pch=1)

#abline

abline(v=c(2,-2), col="red", lwd=3)

abline(h=0.05, col="red",lwd=3)

highlight the genes with color

points(log(fc_sig_results_genes_plot$fc),fc_sig_results_genes_plot$qval, col="green", pch=16)

make the log results to se who is upregulated and downregulated

logresuts= log(fc_sig_results_genes_plot$fc)

label the significant genes

textxy(log(fc_sig_results_genes_plot$fc),fc_sig_results_genes_plot$qval, labs=fc_sig_results_genes_plot$id, cex=1)

write.table(logresuts, "/Workspace/Regulation_Foldchange_controls_treatmnets.txt", sep="/t")

write the results to the file after filtering by p value <0.05, fold change (by 4 fold). Then sort the genes by p-value.

```
# Identify the genes (rows) below p-value 0.05

sigpi = which(results_genes[,"pval"]<0.05)

# Extract p-significant genes in a separate object

sigp = results_genes[sigpi,]

--------------------------------------

--------------------------------------

# Identify the statistically significant genes (rows) that are upregulated/ ##

## downregulated by 4 fold

sigde = which(abs (log(sigp[,"pval"])) >= 2)

# Extract and store the statistically significant genes (rows) that are upregulated/
downregulated by 4 fold

sig_tn_de = sigp[sigde,]

# Order by q value, followed by differential expression

sorted_sig_tn_de = order(sig_tn_de[,"pval"], decreasing=FALSE)

## Extract the columns of interest (gene id, fold change, log fold change, p-value and
adjusted p-value) from the sorted list

# Extract gene expression values using significant genes

sig_gene_expression=gene_expression[rownames(gene_expression) %in%
sig_tn_de$id,]

#remove Sp_controls and Sp_treatments columns

sig_gene_expression=sig_gene_expression[,-c(7:8)]


## Heatmap

# Extract gene expression values using significant genes

sig_gene_expression=gene_expression[rownames(gene_expression) %in%
sig_tn_de$id,]

#remove average columns

sig_gene_expression=sig_gene_expression[,-c(7:8)]
```

for pheatmap function, column names and row names of data and pdata mush be identical# change the row names

rownames(phenotype_table)=phenotype_table[,1]

remove the id column

phenotype_table=subset(phenotype_table, select = -c(id))

change the colnames to match with the sample names

colnames(sig_gene_expression)=row.names(phenotype_table)

draw heatmap

install.packages("pheatmap")

library(pheatmap)

library(RColorBrewer)

pheatmap(as.matrix(sig_gene_expression), scale = "row", clustering_distance_rows = "correlation",

clustering_method = "complete",annotation_col = phenotype_table, annotation_colors = grey,

main="Significant genes",fontsize_col=8, fontsize_row = 4 , col=brewer.pal(11,"Spectral"),

show_rownames = F)

Supplemental script 7: RNA genotyping

##RNA-seq Genotyping

Building NGSEP

tar -xzvf NGSEPcore_4.0.1.tar.gz

cd NGSEPcore_4.0.1

make all

Note: Usage fields below do not include the version number. To remove the

version number, users can either copy the executable jar file:

cp NGSEPcore_4.0.1.jar NGSEPcore.jar

or just make a symbolic link:

ln -s NGSEPcore_4.0.1.jar NGSEPcore.jar

Calling variants over multiple samples

This module allows to call variants over a group of samples separated by files or read group tags. This is now the recommended method to perform variants detection on genotype-by-sequencing (GBS), RAD sequencing, whole exome sequencing (WES), RNA-seq, and low coverage (less than 10x) whole genome sequencing (WGS) data. Although it can also be used on high coverage WGS data, the classic sample-by-sample analysis (commands FindVariants, MergeVariants and MergeVCF) is still recommended to identify structural variants. This module requires one or more read alignment files in SAM or BAM format and the reference genome that was used to produce the alignments.

java -jar NGSEPcore.jar MultisampleVariantsDetector <OPTIONS> <BAM_FILES>*

To select only the central region use the flag -ignore at each end

To select the score quality use the falg -minQuality

java -jar -Xmx32g /NGSEPcore_4.0.1.jar MultisampleVariantsDetector -ignore5 10 -ignore3 10 -minQuality 40 -r /References/Nuclear/GDDH13_1-1_formatted.fasta *.bam >& MultisampleVariantsDetector.log &

Functional annotation of variants

Generates a VCF file including the functional information related to each variant. Requires a gff3 file with gene annotations, and the reference genome in fasta format. Reads from standard input unless the -i option is used to specify an input file. Writes to standard output unless the -o option is used to specify an output file.

java -jar -Xmx32g /NGSEPcore_4.0.1.jar VCFAnnotate -i variants.vcf -r /references.fasta -t /References.gff3 -o variants_annotated.vcf >& variants_annotated.log &

Filtering VCF files

This module implements different filters on VCF files with genotype information and generates a VCF file with variants passing the filtering criteria.

Use -m flag to filter the minimum number of samples genotyped to keep the variant

Use -s flag to keep only biallelic SNPs

java -jar -Xmx32g /NGSEPcore_4.0.1.jar VCFFilter -i variants_annotated.vcf -m 52 -s -o variants_annotated_allgenotyped_OnlySNps.vcf &

Calculating summary statistics

Generate a report with the variants included in a VCF file for different categories.

java -jar -Xmx32g /NGSEPcore_4.0.1.jar VCFSummaryStats -i variants_annotated_allgenotyped_OnlySNPs.vcf -o variants_annotated_allgenotyped_OnlySNPs_Stats.txt &

Convert VCF files to other formats

Convert genotype calls in VCF format to flapjack format to see the samples genotypes

java -jar -Xmx32g /NGSEPcore_4.0.1.jar VCFConverter -i variants_annotated_allgenotyped_OnlySNPs.vcf -flapjack -o variants_annotated_allgenotyped_OnlySNPs &

Building dendograms using the Neighbor-Joining algorithm

Given a distance matrix file, this command builds a dendogram for graphical display of genetic distances using the Neighbor Joining algorithm. The distance matrix can be provided as an upper, lower or full matrix. The dendogram is written in Newick format. Reads from standard input unless the -i option is used to specify an input file. Writes to standard output unless the -o option is used to specify an output file.

java -jar -Xmx32g /NGSEPcore_4.0.1.jar VCFDistanceMatrixCalculator -i variants_annotated_allgenotyped_OnlySNPs.vcf -o variants_annotated_allgenotyped_OnlySNPs_distancematrix &

```
java -jar -Xmx32g /NGSEPcore_4.0.1.jar NeighborJoining -i
variants_annotated_allgenotyped_OnlySNPs_distancematrix.txt -o
variants_annotated_allgenotyped_OnlySNPs_distancematrix_NJ.txt &
```
